# The responses of extracellular enzyme activities and microbial community composition under nitrogen addition in an upland soil

**DOI:** 10.1371/journal.pone.0223026

**Published:** 2019-09-30

**Authors:** Sami Ullah, Chao Ai, Shaohui Huang, Jiajia Zhang, Liangliang Jia, Jinchuan Ma, Wei Zhou, Ping He

**Affiliations:** 1 Ministry of Agriculture Key Laboratory of Plant Nutrition and Fertilizer, Institute of Agricultural Resources and Regional Planning, Chinese Academy of Agricultural Sciences (CAAS), Beijing, PR China; 2 Hebei Academy of Agriculture and Forestry Sciences, Hebei, PR China; RMIT University, AUSTRALIA

## Abstract

Tremendous amounts of nitrogen (N) fertilizer have been added to arable lands, often resulting in substantial effects on terrestrial ecosystems, including soil acidification, altered enzyme activities and changes in microbial community composition. Soil microbes are the major drivers of soil carbon (C) and N cycling; therefore, understanding the response of microbial communities to elevated N inputs is of significant importance. This study was carried out to investigate the influences of different N fertilization rates (0, 182, and 225 kg ha^-1^ representing control, low, and high N supply for each crop season for summer maize and winter wheat) on soil biochemical attributes, extracellular enzyme activities, and the microbial community composition in a winter wheat-summer maize rotation cropping system in north-central China. The results showed that N addition significantly decreased the soil pH in both the wheat and maize seasons. Microbial biomass N (MBN) decreased following N fertilization in the wheat season, while the opposite trend in MBN was observed in the maize season. Response ratio analysis showed that the activities of enzymes involved in C, N, and phosphorus cycling were significantly enhanced under N enrichment in both the wheat and maize seasons, and higher enzyme activities were noted in the high N addition treatment than in the low N addition treatment. A linear increase in fungal abundance with the N addition gradient was observed in the wheat season, whereas the fungal abundance increased and then decreased in the maize season. The bacterial abundance showed an increased and then decreased trend in response to the N addition gradient in both the wheat and maize crop seasons. Moreover, the partial least squares path model (PLS-PM) analysis showed that soil pH and soil organic carbon (SOC) were the most important soil variables, causing shifts in the soil bacteria. Furthermore, compared with the N-cycling enzymes, the C-cycling enzymes were significantly affected by the soil pH and SOC. Taken together, these results suggest that the effect of N addition on enzyme activities was consistent in both crop seasons, while the effects on MBN and microbial community composition to N addition were highly variable in the two crop seasons. Moreover, N fertilization-induced changes in the soil chemical properties such as soil acidity and SOC played a substantial role in shaping the microbial community.

## Introduction

The global demand for nitrogen (N) fertilizer for crop production is increasing daily, and as a result, the global N fertilizer demand is expected to increase from 86 Tg (860,0000 metric tons) of N in 1995 to 135 Tg of N in 2050 [1,2]. The overuse of N fertilization in crop production is practiced in almost every country worldwide, and approximately 50% of the N that is applied is lost to the ecosphere, with widespread implications [3]. Recent evidence suggests that excessive N enrichment contributes to a number of deleterious environmental outcomes. For example, N can alter the quality and quantity of soil organic matter (SOM), deplete soil nutrients and acidify soils, and all of these changes negatively impact belowground microbial diversity and community composition, which drive nutrient cycling in arable lands [4–6]. To date, numerous studies have been conducted to evaluate the response of the microbial community composition under N addition [6–9]. However, the response of belowground microbiota to anthropogenic N enrichment remains inconsistent. For instance, some researchers demonstrated that N enrichment increased microbial abundance, decreased diversity, and altered the microbial community structure [6, 10], and N input has been identified as a key factor impacting microbial community composition in terrestrial ecosystems. In contrast, Zhao et al. (2014) demonstrated that N addition did not increase microbial biomass; additionally, N fertilization did increase the abundances of bacterial [11] and fungal communities [12]. Furthermore, it remains unclear how N addition governs microbial biomass C and N (MBC and MBN) dynamics. In a meta-analysis, Treseder (2008) found that N addition increased MBC. In contrast, another meta-analysis suggested that N addition decreased MBC [13]. Furthermore, it was reported that microbial biomass was not affected as a result of N addition [14]. Moreover, a study demonstrated that MBN increased following N addition [15]. Alternatively, Wang et al. (2018a) found that MBN decreased as a result of N fertilization. These divergent results demonstrate that how N enrichment governs the microbial C and N pools and community structure in terrestrial ecosystems remains unclear.

Experimental evidence has shown that to fulfill nutrient and energy demands, soil microorganisms can secrete extracellular enzymes to break down polymerized SOM into assimilable small molecules [16]. Numerous extracellular enzyme activities are involved in N and C turnover [17, 18]. Generally, extracellular enzymes consist of cellulases, oxidases and hydrolases that break down substrates of different complexities and compositions [19]. Cellulases are hydrolytic enzymes that are produced by soil microbes during the breakdown of polysaccharides; they include β-glucosidase, β-xylosidase, and β-cellobiosidase [19]. The enzymes produced by microbes during N cycling include urease, leucine amino peptidase, and N-acetyl-glucosaminidase, which target urea, protein, and chitin, respectively [17]. Earlier reports indicated that enzyme activities are influenced by N fertilization. For instance, a meta-analysis revealed that N fertilization increased hydrolases but decreased oxidases [20].

Studies have demonstrated that N addition impacts microbial community structure by altering soil chemical properties. For instance, researchers pointed out that changes in soil pH can cause ambient shifts in microbial groups [18, 21]. Additionally, variations in soil total N (TN), soil organic carbon (SOC), and NO_3_^-^ N levels as a result of N enrichment can also impact the soil microbial population [6, 11, 18]. Likewise, N addition-induced changes in soil properties can impact enzyme activities [6, 18]. However, various groups of enzymes may respond distinctly to soil variables. For example, Dai et al. (2019) concluded that the soil carbon:nitrogen (C:N) ratio and pH together significantly affected C-cycling enzymes but not N-cycling enzymes. Moreover, shifts in microbial community structure depend not only on N addition but also on plant type since plant type can profoundly impact the adjoining soil and its microbiota [22]. A study demonstrated that more carbon was released as a result of respiration from the roots of wheat than from those of maize [23]. Thus, the different patterns of N mineralization and the distributions of N sources between the two crops possibly contribute to partial changes in microbial groups [24]. Therefore, exploring the relationship between soil variables and microbial community composition and enzyme activities is imperative for understanding the impact of N enrichment under different crop types on soil biological activity and function.

In north-central China, the winter wheat and summer maize rotation cropping system is intensively cultivated, accounting for approximately 61% and 39% of wheat and maize production, respectively [25]. Sustainable crop production is greatly needed in this major grain-producing region to fulfill China’s substantial food demand. In pursuit of higher crop yields, Chinese farmers apply tremendous amounts of fertilizers, resulting in substantial environmental effects [26, 27] and alterations to the microbial community composition [7]. Therefore, it is highly imperative to study how soil chemical properties, the microbial community composition, and enzyme activities respond to elevated N inputs. We addressed these questions in a long-term N fertilization field trial in an intensively managed winter wheat-summer maize cropping system in north-central China. The objectives of the present study were to (i) examine the response of MBN and MBC under long-term N fertilization, (ii) identify the changes in soil enzyme activities and microbial community composition in response to N fertilization and (iii) identify important soil variables influencing soil enzyme activities and microbial communities. We hypothesized that (i) a distinct response of MBN would be observed in the two crop seasons because crop type and season can cause shifts in microbial biomass [28]; (ii) changes in the soil chemical attributes and microbial community would significantly enhance the enzyme activities related to C and P cycling but decrease the enzyme activities related to N and phenolic compound oxidase C cycling because of potential decreases in the availability of C and P but improvement to the N condition through amendment [29]; and (iii) among other soil chemical attributes, soil pH and SOC would be the key indicators for evaluating the impact of long-term N addition on the soil microbial communities and enzyme activities.

## Materials and methods

### Site description

The field trial was established in 2009 at the Dahe experimental station in Shijiazhuang city, Hebei Province (38°07ʹ N and 114°29ʹ E), north-central China. The wheat-maize rotation system is prevalent in the experimental region. The study area has a typical warm temperate and subhumid continental monsoon climate. The average annual temperature is 14.3 °C, and precipitation is 400 mm. The study area has fluvo-aquic soil (Calcaric Cambisols, FAO) [30]. Initial soil fertility before the experiment was pH of 8.1, soil organic carbon of 17.5 g kg^-1^, total N of 1.14 g kg^-1^, available P of 13.63 mg kg^-1^, and available K of 96.56 mg kg^-1^.

### Experimental design

The experiment was conducted in a randomized complete block design with four replicates, and the plot size was 45 m^2^ (5 m × 9 m). There were three treatments in each crop season: control (No N added), low addition (182 kg ha^-1^ of N), and high addition (225 kg ha^-1^ of N). Thus, in one year, no N was added, 364 kg ha^-1^ of N was added, and 450 kg ha^-1^ of N was added. In total, there were 12 experimental plots (3 treatments × 4 replicates) and the same experimental plots were used for both the winter wheat and summer maize crops. The high N rate i.e. 225 kg ha^-1^ is common N fertilizer rate applied in Hebei province of China. The other is low N rate i.e. 182 kg ha^-1^ where N rate is 19% less than the N fertilizer rate applied in Hebei province of China. The source of N fertilizer in our study was urea. Half of the N was applied as basal fertilizer, and the remaining N was applied at the wheat jointing stage and at the maize tasseling stage. No organic manure or compost was applied. After harvesting the wheat and maize in June and October 2017, respectively, five soil cores (2 cm in diameter) were collected from each experimental plot to a depth of 0–20 cm and pooled to create a composite sample. One part of the subsample was kept at -80 °C for further molecular analysis.

### Chemical analysis

The SOC and TN concentrations were analyzed by dichromate oxidation [31] and Kjeldahl digestion, respectively [32]. The soil inorganic nitrogen (NO_3_^-^ N and NH_4_^+^ N) content was measured with 12 g of a fresh soil sample using a 1:10 ratio of soil to a 0.01 mol L^-1^ CaCl_2_ solution and then analyzed using continuous flow analysis (Foss FlAstar 5000, Sweden). The soil pH was measured with a compound electrode (PE-10, Sartorius, Germany) using a soil:water ratio of 1:2.5. The MBC and MBN analyses were carried out with the fumigation extraction method with 0.5 M K_2_SO_4_ [33] and determined with a total organic C/N analyzer (Multi N/C3100/HT1300, Analytik Jena AG).

### Extracellular enzyme activities

Detailed information on the enzymes involved in N, carbon (C), phosphorus (P), sulfur (S), and phenolic compound oxidase cycling is shown in Table 1. The activities of extracellular enzymes associated with N, C, P and S were determined according to the fluorescence-based protocols described in [11] and expressed in units of nmol h^-1^g^-1^. In brief, 1 g of fresh soil was homogenized in 100 mL of sterilized water using a polytron homogenizer. Then, a magnetic stirrer was used to maintain a uniform suspension. The sample suspension, sterilized water, 200 μM of 4-methylumbelliferyl-linked substrates, and 10 μM of references were added into the wells of a black 96-well microplate. The microplates were covered and incubated in the dark at 25 °C for 4 h. After incubation, 10 μL of a 1 M NaOH solution was added rapidly to each well of the microplate to stop the enzymatic reaction. Fluorescence was quantified using a microplate fluorometer (Scientific Fluoroskan Ascent FL, Thermo Fisher Scientific, Waltham, MA, USA) with 365 nm excitation and 450 nm emission filters. Phenol oxidase (PO) and peroxidase (PEO) were quantified colorimetrically in a clear 96-well microplate as described in [11].

**Table 1:** Extracellular enzymes assayed in both the wheat and maize season soil, their enzyme commission number (EC) and corresponding substrate (_L_-DOPA = L-3,4-dihydroxyphenylalanine, 4-MUB = 4-methylumbelliferyl).

Nutrient cycle	Enzyme	Abbreviation	Substrate	EC
N cycle	α-glucosidase	AG	4-MUB-α-_D_-glucoside	3.2.1.20
	L-leucine aminopeptidase	LAP	_L_-Leucine-7-amino-4-methylcoumarin	3.4.11.1
	N-acetyl-β-glucosaminidase	NAG	4-MUB-N-acetyl-β-_D_-glucosaminide	3.2.1.30
C cycle	β-glucosidase	BG	4-MUB-β-_D_-glucoside	3.2.1.21
	β-cellobiosidase	BC	4-MUB-β-_D_-cellobioside	3.2.1.91
	β-xylosidase	BX	4-MUB-β-_D_-xyloside	3.2.1.37
P cycle	Phosphatase	PHOS	4-MUB-phosphate	3.1.3.1
S cycle	Sulfatase	SUL	4-MUB-sulfate	3.1.6.1
Phenolic compounds oxidase	Phenol oxidase	PO	_L_-DOPA	1.10.3.2
	Peroxidase	PEO	_L_-DOPA	1.11.1.7

### Phospholipid fatty acid (PLFA) analysis

The soil microbial community and microbial biomass were quantified with PLFA analysis as previously described in [34]. Briefly, the PLFAs were extracted from three grams of freeze-dried soil samples using a chloroform/methanol/citric acid buffer (1:2:0.8 volume ratio, pH 4.0). Glycolipids and neutral lipids were separated from polar lipids on a silica-bonded phase column (SPE-Si, Supelco, Poole, UK) by elution with acetone and chloroform, respectively. Nonadecanoic acid methy ester (19:0) was added as an internal standard, and the polar lipids were then converted to fatty acid methyl esters (FAMEs) by mild alkaline methanolysis. The dried FAMEs were dissolved again in n-hexane and then measured and identified with the MIDI Sherlock microbial identification system version 4.5 (MIDI Inc., Newark, DE, USA) and gas chromatography (N6890, Agilent). The concentrations of PLFAs were expressed as nmol g^-1^ dry soil. The total microbial biomass was measured using the total concentration of PLFAs (nmol g^-1^), and the abundance of each PLFA was determined by its % mole abundance in each sample. Individual PLFAs, including bacteria, gram-positive (G+) and gram-negative (G-) bacteria, fungi, arbuscular mycorrhizal fungi (AMF), and actinomycete, were used as signatures for the various taxonomic groups of microorganisms according to previously published fatty acid biomarker data [11, 18, 35] and are shown in Table 2.

**Table 2:** Phospholipid fatty acids used as signature biomarkers.

	Microbial groups					
	General bacteria	Gram^+^ bacteria	Gram^-^ bacteria	Fungi	Arbuscular mycorrhizal fungi	Actinomycete
PLFA biomarkers	14:0, 15:0, 16:0, 17:0	i 14:0, i 15:0, i 16:0, i 17:0, i 18:0	16:1 w7c, 17:1 w8c, 18:1 w5c, 18:1 w7c	18:2 w6c, 18:2 w9c	16:1w5c	16:0 (10Me), 17:0 (10Me), 18:0 (10Me)

Note: Throughout the manuscript bacterial PLFAs means the sum of Gram^+^ and Gram^-^ biomarkers together with general bacteria biomarkers.

### Statistical analyses

For each variable that was measured, the data were analyzed by one-way analysis of variance (ANOVA) using Fisher’s least significant difference (LSD) at P ≤ 0.05 using SPSS software version 24 (SPSS Inc., Chicago, IL, USA). The variables such as pH, TN, NO3^-^, NH4^+^, SOC, total PLFA’s, relative abundance of bacteria, fungi, G+, G-, actinomycete, and AMF were selected as fixed effects. To separate the effects of N addition and cropping season on soil extracellular enzyme activities and microbial community composition, we used permutational multivariate analysis of variance (PERMANOVA) (Monte Carlo, 999 permutations) using Primer software version 6.0 (Plymouth, UK). The data was transformed to square root before running PERMANOVA. We used Bray-Curtis distance matrix for both enzyme activities and microbial community composition in conducting PERMANOVA analysis. Response ratio analysis was used to reveal the changes in enzyme activities between the control and N addition treatments with 95% confidence intervals [36]. Meta-analysis was used to combine the results of multiple independent studies or multiple independent variables within a single study. This type of analysis has been successfully applied in single studies of the responses of extracellular enzymes to N amendments [37]. Principal coordinate analysis (PCoA) was used to identify changes in the activities of enzymes and shifts in the microbial community structure as a result of N addition using Primer software version 6.0. We explored the relationships among soil chemical properties (pH, SOC, TN, and NO_3_^-^ N), bacterial and fungal PLFAs and soil enzymes (C-cycling enzymes and N-cycling enzymes) under N fertilization by using partial least squares path modeling (PLS-PM), an important statistical tool that can demonstrate cause and effect relationships among observed and latent variables [38]. The path coefficients (i.e., direct effects) were the strength and direction of the linear relationships between the variables (the relationship between the soil properties with bacteria, fungi, and enzyme activities); the indirect effects indicated the existence of multiple path coefficients between a predictor and a response variable, and the products of all possible paths were summed except for the direct effect [39]. The estimates of path coefficients and the coefficients of determination (R^2^) were validated by R (v. 3.4.4) using the plspm package (1000 bootstraps).

## Results

### Soil biochemical properties

**Fig 1:**
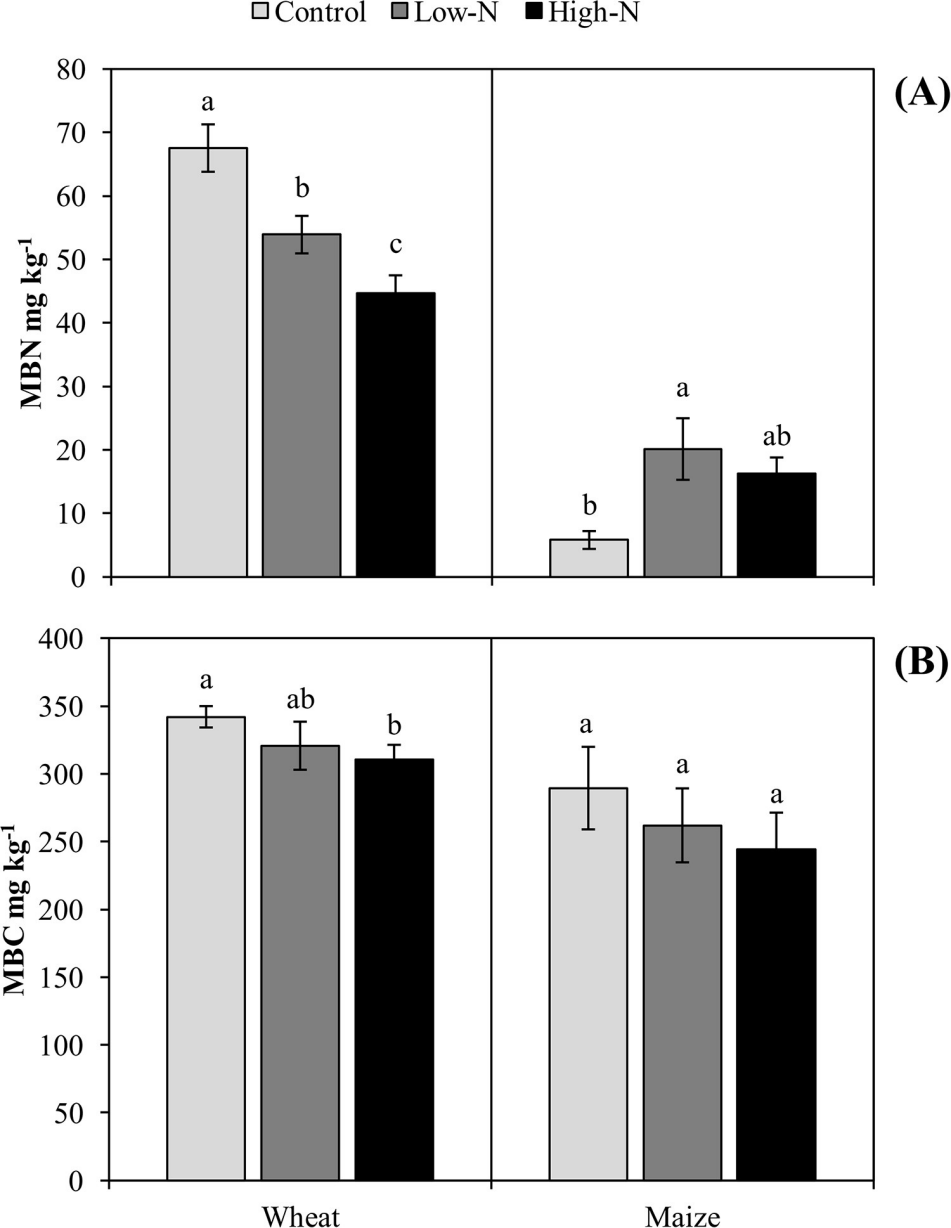
Effect of long-term N fertilization on, microbial biomass nitrogen (MBN) (A), and microbial biomass carbon (MBC) (B). Data are the means (n = 4) and error bars indicates standard error. Different letters indicate significant differences among treatments at P ≤ 0.05 as determined by LSD.

N fertilization significantly changed the soil chemical properties (Table 3). In each season, N fertilization significantly increased the TN, NO_3_^-^ N, and SOC levels compared with those in the control. The highest TN and NO_3_^-^ N stocks were found in the high N treatment, while the highest SOC content was recorded in the low N treatment. However, the soil pH in each season decreased following N enrichment, and the lowest soil pH was found in the high N treatment. There was no significant effect of the various N fertilization rates on the NH_4_^+^ N content in the two crop seasons. Moreover, the MBN content during the wheat season rapidly declined due to N addition, and the lowest MBN level was recorded in the high N treatment (Fig 1A). Interestingly, in the maize season, the opposite trend was noted, and the highest MBN content was found in the low N treatment. N fertilization decreased the MBC in the two crop soils (Fig 1B).

**Table 3:** Effect of long-term N fertilization on soil properties.

	pH	TN^a^ (g kg^-1^)	NO_3_^-^ N (mg kg^-1^)	NH_4_^+^ N (mg kg^-1^)	SOC^a^ (g kg^-1^)	C:N
Wheat season						
Control	8.12 ± 0.017 a^b^	1.19 ± 0.013 c	5.86 ± 0.87 b	3.22 ± 0.64 a	20.57 ± 0.55 b	17.35 ± a 0.37 a
Low-N	8.00 ± 0.018 b	1.28 ± 0.019 b	11.07 ± 1.37 ab	2.81 ± 0.33 a	22.11 ± 0.18 a	17.32 ± a 0.16 a
High-N	7.91 ± 0.016 c	1.32 ± 0.015 a	19.86 ± 5.08 a	2.69 ± 0.06 a	21.97 ± 0.20 a	16.69 ± a 0.24 a
Source of variation						
df	2	2	2	2	2	2
SS	0.08	0.03	400	0.60	5.70	1.48
F	26.22	33.49	6.01	0.33	12.97	2.12
*P*	≤ 0.001	≤ 0.001	≤ 0.05	0.68	≤ 0.01	0.20
Maize season						
Control	7.96 ± 0.028 a	1.15 ± 0.016 b	3.06 ± 0.37 b	1.54 ± 0.23 a	18.53 ± 0.45 b	16.43 ± a 0.18 a
Low-N	7.79 ± 0.028 b	1.24 ± 0.024 a	14.65 ± 1.13 a	1.79 ± 0.50 a	20.28 ± 0.57 a	16.38 ± a 0.26 a
High-N	7.80 ± 0.006 b	1.27 ±0.016 a	14.87 ± 1.06 a	1.29 ± 0.12 a	20.73 ±0.32 a	16.33 ± a 0.08 a
Source of variation						
df	2	2	2	2	2	2
SS	0.07	0.03	364	0.49	10.75	0.02
F	29.78	9.28	40.87	0.65	8.69	0.07
*P*	≤ 0.001	≤ 0.01	≤ 0.001	0.44	≤ 0.01	0.93

Data are the means ± standard error (n = 4).

^**a**^ TN = total nitrogen, SOC = soil organic carbon.

^**b**^ Different letters in a column indicate significant differences among treatments at *P* ≤ 0.05 as determined by fisher’s least significant difference (LSD). Control (No N added), Low (182 kg ha^-1^ of N), and High additions (225 kg ha^-1^ of N) in each crop season.

### Extracellular enzyme activities

**Fig 2:**
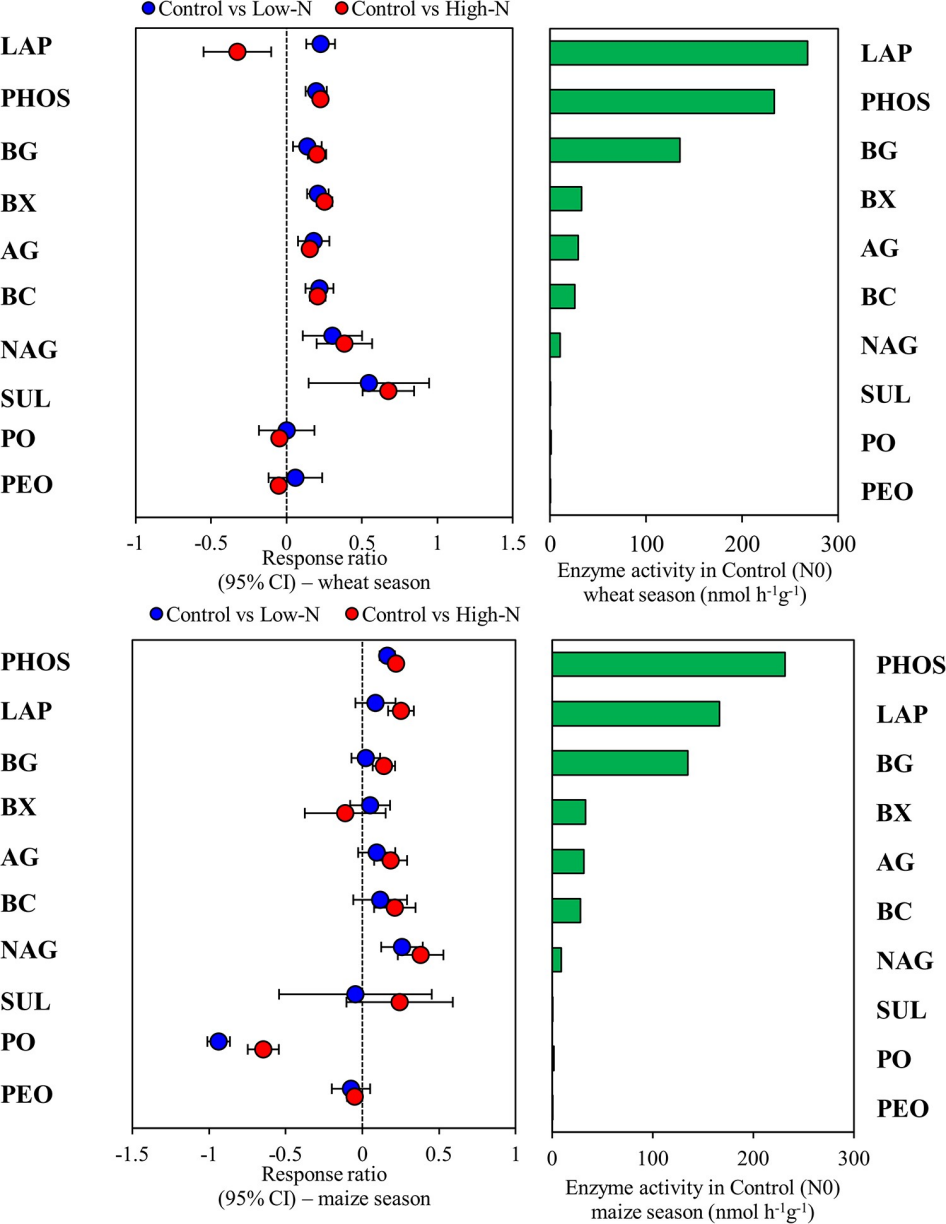
Significantly altered enzyme activities in wheat and maize season following N fertilization as measured by the response ratio method at the 95% confidence interval. Error bar symbols plotted to the right of the dashed line indicates that the enzyme activities increased, while those at the left side decreased. See Table 1 for abbreviations. Horizontal bars indicates enzyme activities in control.

**Fig 3:**
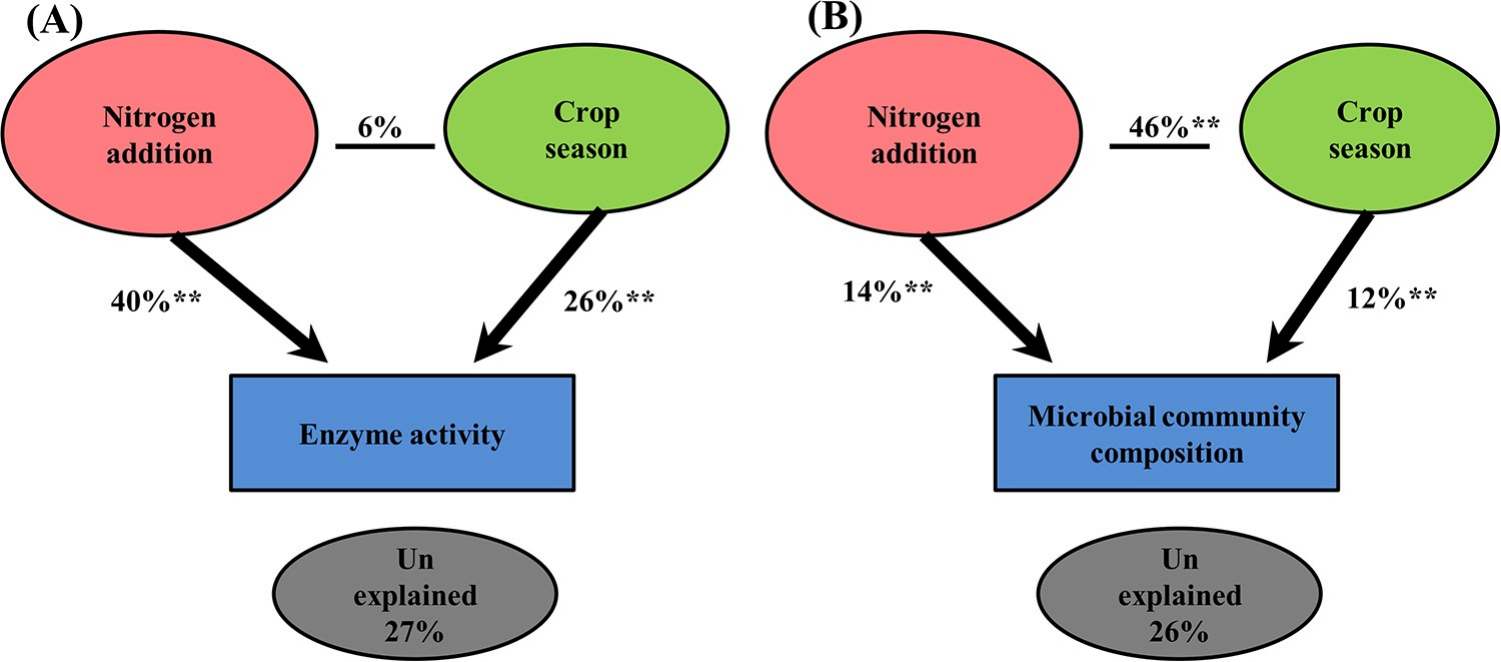
PERMANOVA values showing the percent that N fertilization and crop season contributed to the variation in enzyme activities (A) and microbial community composition (B). * P ≤ 0.01, ** P ≤ 0.001.

**Fig 4:**
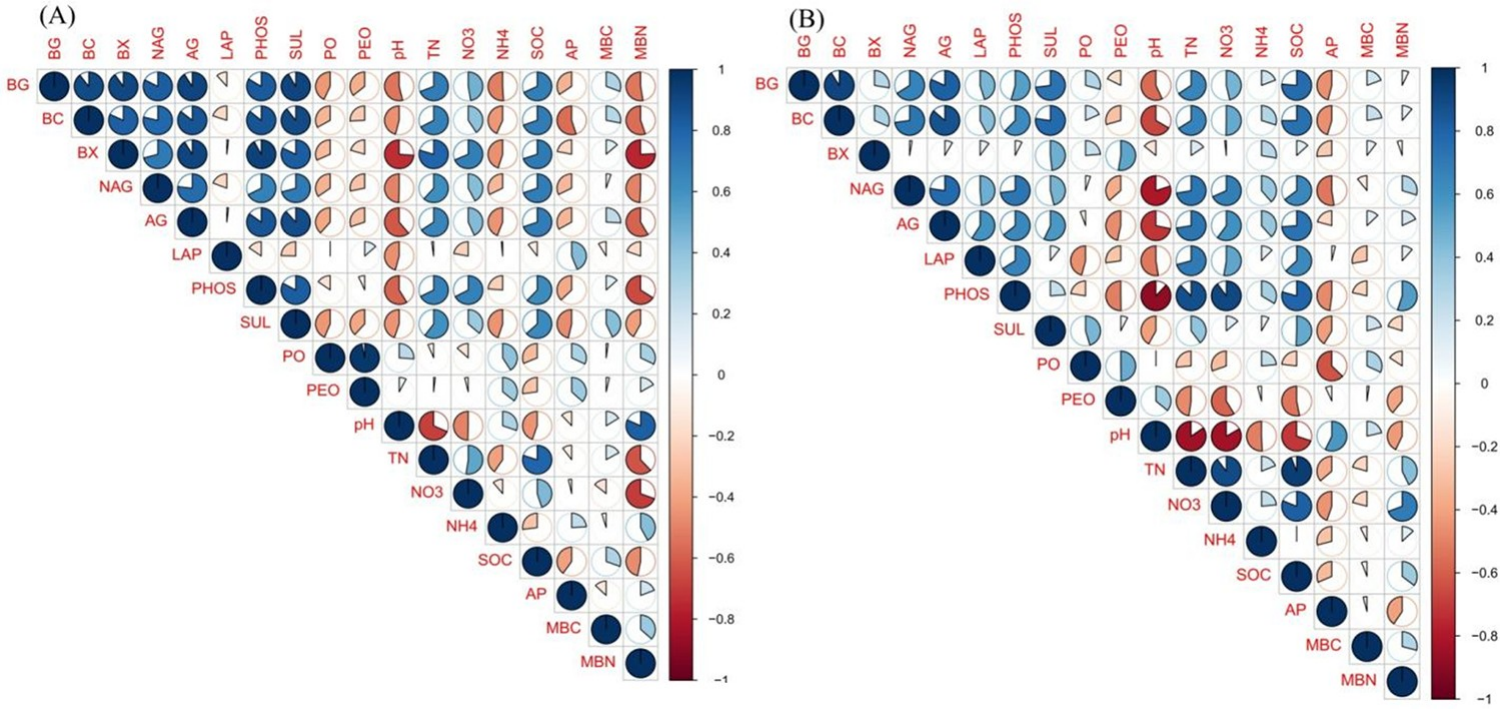
Pearson correlation analysis between soil properties and enzymes of wheat (A) and maize season (B). Red and blue circles represent negative and positive correlation, respectively. The extent of correlation is indicated by pie fill area, i.e., larger to smaller pie fill area indicates high to low correlation. See Table1, Table 3 and Fig 1 for enzyme activities, soil chemical properties and biological traits abbreviations respectively.

We used response ratio analysis to reveal the impact of N fertilization on enzyme activities. In the wheat season, compared with the results in the control treatment, the low N and high N treatments significantly increased the activities of phosphatase, sulfatase, α-glucosidase, N-acetyl-glucosaminidase, β-cellobiosidase, β-glucosidase, and β-xylosidase (Fig 2). The activities of phenol oxidase and peroxidase were not affected by the low N treatment, but their activities significantly declined in the high N treatment. In the maize season, both the low N and high N rate treatments significantly enhanced the phosphatase and N-acetyl-glucosaminidase activities but depressed phenol oxidase activities compared to those in the control (Fig 2). The leucine amino peptidase, α-glucosidase, β-glucosidase, β-cellobiosidase, and β-xylosidase enzyme activities were slightly affected by the low N treatment; however, the high N treatment significantly increased the leucine amino peptidase, α-glucosidase, β-glucosidase, β-cellobiosidase, and β-xylosidase enzyme activities. To further evaluate the effects of N fertilization and crop season on extracellular enzyme activities, we conducted PCO analysis. As shown in the results, PCO1 explained the majority of the variation, 49.5%, while PCO2 explained 45.6% of the variation (S1 Fig). Moreover, PERMANOVA showed that N addition had greater (40%) impacts on enzyme activity, followed by crop season (26%) (Fig 3A; S1 Table). We further found that the enzymes involved in C, N, and P cycling were strongly correlated with each other; for instance, the β-glucosidase and β-cellobiosidase activities were strongly correlated with the N-acetyl-glucosaminidase and phosphatase activities (Fig 4). Overall, the majority of the enzyme activities were negatively correlated with the soil pH but positively correlated with the TN and SOC levels.

### Microbial community composition

In the wheat season, compared with the levels in the control, the low N and high N treatments greatly increased the total PLFAs, and the highest PLFA abundance was found in the high N treatment (Table 4). However, there was no significant effect in either the low N or high N treatments regarding the relative abundances of bacteria, fungi, and actinomycete PLFAs. In the maize season, the highest abundance of total PLFAs was recorded in the low N treatment. There was a slight effect of N addition on the relative abundances of the bacterial and fungal PLFAs. N addition significantly reduced the abundance of AMF. To further explore the effect of N fertilization and crop season on the microbial community composition, we conducted PCO analysis. The PCO results showed that PCO1 accounted for the majority of the variation, 70.6%, and PCO2 accounted for 11.8% of the variation (S2 Fig). Moreover, PERMANOVA revealed that N addition had a greater (14%) impact on the microbial community composition than crop season (12%). The interaction between N addition and crop type explained 46% of the variation (Fig 3B; S2 Table).

**Table 4:** The total PLFAs and the relative abundance of the individual PLFAs (mol %) in soil samples from the upland soil.

Wheat	Total PLFAs^a^	Bacteria (mol %)	G+^a^ (mol %)	G-^a^ (mol %)	Fungi (mol %)	Act^a^ (mol %)	AMF^a^ (mol %)
Wheat season							
Control	60.33 ± 2.36 b^b^	51.56 ± 2.89 a	18.29 ± 0.41 a	14.89 ± 2.83 a	11.01 ± 0.27 a	11.86 ± 0.10 a	3.98 ± 0.06 a
Low-N	75.88 ± 1.97 a	54.98 ± 0.42 a	17.92 ± 0.04 a	18.81 ± 0.17 a	10.58 ± 0.15 a	11.97 ± 0.26 a	3.64 ± 0.05 b
High-N	81.68 ± 3.55 a	54.42 ± 0.21 a	16.83 ± 1.28 a	19.03 ± 0.18 a	10.81 ± 0.50 a	11.90 ± 0.19 a	3.84 ± 0.04 ab
Source of variation							
df	02	02	02	02	02	02	02
SS	975	26.92	4.61	43.31	0.10	0.02	0.23
F	22.13	1.20	0.79	1.99	0.18	0.09	6.43
*P*	≤ 0.05	0.36	0.49	0.21	0.83	0.91	≤ 0.05
Maize season							
Control	66.33 ± 4.05 a	52.74 ± 0.20 a	17.56 ± 0.41 a	17.42 ± 0.23 a	11.55 ± 0.79 a	11.64 ± 0.21 a	3.80 ± 0.12 a
Low-N	73.81 ± 2.78 a	53.60 ± 0.38 a	16.95 ± 1.22 a	17.50 ± 0.16 a	10.67 ± 0.35 a	12.24 ± 0.12 a	3.42 ± 0.05 b
High-N	44.71 ± 3.68 b	53.48 ± 0.69 a	18.05 ± 0.52 a	17.06 ± 0.44 a	10.04 ± 0.46 a	11.67 ± 0.24 a	3.07 ± 0.11 c
Source of variation							
df	02	02	02	02	02	02	02
SS	1827	1.72	2.43	0.44	3.31	0.90	1.08
F	45.20	0.78	0.32	0.63	1.53	4.07	14.30
*P*	≤ 0.001	0.49	0.73	0.53	0.28	0.07	≤ 0.001

Data are the means ± standard error (n = 4). Different letters indicate significant differences among treatments at *P* ≤ 0.05 as determined by LSD.

^**a**^ PLFAs = phospholipid fatty acids. G+ = gram positive, G- = gram negative, Act = actinomycete, AMF = arbuscular mycorrhizal fungi

^**b**^ Different letters in a column indicate significant differences among treatments at P ≤ 0.05 as determined by fisher’s least significant difference (LSD).

### PLS-PM

**Fig 5:**
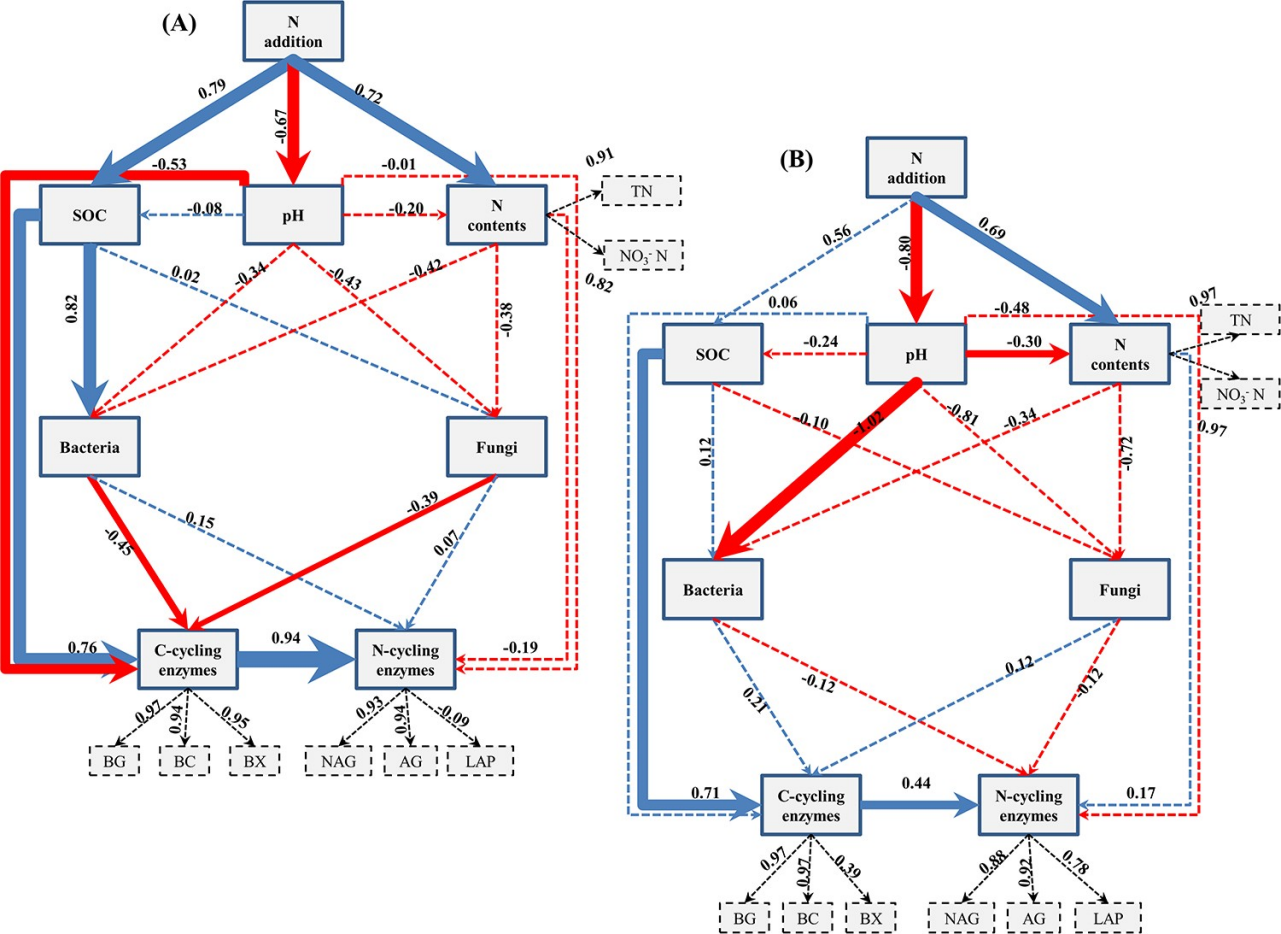
Directed graph of the partial least squares path model (PLS-PM) for wheat (A) and maize season (B). Each box represents an observed variable (i.e., measured) or latent variable (i.e., constructs). The loading for N contents and 6 enzyme activities that create the latent variables are shown in the dashed rectangle. Path coefficients showing the relationship between soil properties with bacteria, fungi, and EEAs are calculated after 1000 bootstraps and reflected in the width of the arrow, with blue and red indicating positive and negative effects, respectively. Dashed arrows show that coefficients did not differ significantly from 0 (P ≥ 0.05). The model is assessed using the Goodness of Fit (GoF) statistic, and the GoF values were 0.67 and 0.68 in wheat and maize season’s soil respectively.

PLS-PM was used to identify the relationships among N fertilization, soil chemistry, enzyme activities and the soil bacterial and fungal communities. In the wheat season soil, the results revealed that N fertilization had a significant direct positive relationship with the soil N content (0.72) (a combination of the TN and NO_3_^-^ N levels) and the SOC (0.79) content. N addition had a significant negative relationship with the soil pH value (-0.67) (Fig 5A; S3 Table). Additionally, the soil pH had a direct negative but non-significant relationship with the soil bacterial (-0.34) and fungal PLFAs (-0.43). In addition, the SOC content had a direct significant positive relationship (0.82) with the bacterial community but no significant relationship (0.02) with the fungal community. Notably, the soil pH, SOC content and bacterial and fungal communities had a significant direct impact on the activities of C-cycling enzymes (a combination of β-glucosidase, β-cellobiosidase, and β-xylosidase). Furthermore, the N-cycling enzymes (a combination of α-glucosidase, L-leucine aminopeptidase, and N-acetyl-β-glucosaminidase) were influenced by the C-cycling enzymes (1.04). In the maize season soil, the results showed that N fertilization had a significant direct positive relationship with the soil N content (0.69), and the opposite trend was found for soil pH (-0.80) (Fig 5B). The soil pH had a significant direct negative relationship with the bacterial community (-1.02) but no significant relationship (-0.81) with the fungal community. Furthermore, the SOC content had a significant direct positive relationship (0.71) with the activities of the C-cycling enzymes. Moreover, the N-cycling enzymes were influenced by the C-cycling enzymes (0.44).

## Discussion

### N addition effects on soil biochemical properties

Our results showed that the MBN was significantly altered under N fertilization. We noticed a distinct response of MBN in the two crop growing seasons in response to N addition, i.e., a decrease in the wheat season and an increase in the maize season (Fig 1A). The increase in MBN following N fertilization during the maize season was possibly due to the increased immobilization of inorganic N (NO_3_^-^ and NH_4_^+^). Moreover, enhanced MBN contents under N addition can also be explained by several mechanisms, such as soil microbiota coupled with enhanced N contents in tissues, fast turnover, high growth rates, and the production of N-rich extracellular enzymes; such microbiotal mechanisms can lead to enhanced MBN contents [40,41]. In addition, soil microbes are capable of taking up resources in abundance and stocking them in various forms, such as polyphosphates and glycogen, leading to variations in microbial biomass [42]. Another study showed that microbial biomass can act as a short-term sink for available nitrogen and that N enrichment can increase MBN [43]. On the other hand, decreased MBN following N enrichment is possibly due to the accumulation of toxic osmotic potential due to fertilization, which may lead to the suppression of microbial biomass or activity [9]. Furthermore, earlier studies also showed distinct seasonal patterns in MBN dynamics [28]. We further observed that the MBN and MBC contents were higher overall in the wheat season than in the maize season, which could be ascribed to (i) a higher nutrient status in the wheat season than in the maize season (Table 3). It is generally believed that the microbial biomass size is greatly impacted by the soil nutrient level; with more available nutrients, more biomass will be produced [44]. (ii) Differences in soil moisture and soil temperature in the wheat and maize growing seasons could also have been factors [45]. Soil temperature is a key factor for microbial biomass determination because temperature regulates microbial activity; thus, soil temperature was positively linked with soil microbial biomass [46]. MBC and MBN mediate the transformation of bio-available C and N between organic and inorganic forms. Although microbial biomass values represent only a small portion of the total N and C in soils, this small living portion contains a significant amount of nutrients that are required for crops. Thus, microbial biomass has a critical role in soil fertility and nutrient cycling [47]. It is worth noting that compared with the effect in the low N addition treatment, there were significant reductions in MBN and MBC in the high N addition treatment, implying that elevated N inputs have negative effects on MBN and MBC pools.

After 9 years of continuous fertilization, we found that both N addition treatments significantly reduced the soil pH; however, the soil pH reduction was more pronounced in the high N addition treatment (Table 3). Studies have indicated that short-term N addition may temporarily increase the soil pH, but long-term N addition reduced the soil pH [48]. A study based on 10 long-term field studies in China showed that the soil pH levels of plots that were supplied with N fertilizer were reduced by 0.45–2.20 units; alternatively, no reductions in soil pH were seen in the plots without N input [4]. Soil acidification affects plant nutrient availability and inhibits SOM decomposition [8, 49]. Therefore, to maintain soil quality and minimize the environmental impacts of agriculture while simultaneously sustaining high crop productivity, there is an urgent need to reduce fertilizer rates, particularly N fertilizer. To that end, the government of China has launched a grand initiative, Zero Growth of Chemical Fertilizer Use by 2020 [50]. It is expected that such initiatives will not only help sustain high crop productivity and enhance nutrient use efficiency but also protect the environment from the adverse effects of agriculture.

### Carbon-cycling enzyme activity is more susceptible to soil pH and SOC than nitrogen-cycling enzyme activity

The soil enzymes produced by microorganisms play a substantial role in N and C turnover [17]. Our results and numerous studies have indicated that N-induced changes in soil chemical properties affect enzyme activities [6, 20]. In this study, C-cycling enzymes were highly related to SOC and soil pH (Fig 5). The decomposition of SOM induces microbial activity and stimulates the production of enzymes [51]. A reduced C:N ratio results in an enhanced soil N content due to the acceleration of N mineralization, and these processes eventually impact C-cycling enzymes [52]. Numerous studies have indicated that enzyme activities are highly influenced by soil pH [6, 21]. In our study, C-cycling enzymes were significantly affected by soil pH; this result agrees with a recent study showing that C-cycling enzymes were significantly influenced by soil pH rather than N-cycling enzymes [18]. The reason why only the C-cycling enzymes were influenced by soil pH in our study may be because various soil enzymes have different optimal soil pH ranges [53]. Additionally, soil pH has a direct relationship with the rates of enzyme activities engaged in biochemical processes [54]. After 9 years of N enrichment, hydrolases were significantly enhanced, while oxidases declined (Fig 2). Enhanced enzyme activities in plots with N addition indicated that the enzymatic activity of microbes was notable under high N conditions [55]. N addition generally increases the activities of enzymes such as glycosidases (β-cellobiosidase, β-glucosidase, β-xylosidase, and α-glucosidase) in a wide range of ecosystems [20], and these activities are associated with the breakdown of storage carbohydrates, chitin, and cellulose and N mineralization [37]. Although N enrichment remarkably enhanced the soil available N, the N- acquiring enzymes, such as N-acetyl-β-glucosaminidase, also increased. This finding could be due to the fact that N-acetyl-β-glucosaminidase activity may reflect factors other than N demand, i.e., fungal activity and biomass [56]. Moreover, other studies have also shown enhanced N-acetyl-β-glucosaminidase activity following N addition [6, 37]. Further, we found that phosphatase activities increased following N addition. Phosphatase production requires enhanced N levels [57], and the addition of N may have promoted soil microbes to produce more phosphatase. Furthermore, soil acidification as a result of N enrichment could increase the binding of P to mineral soil and restrict the availability of P to soil microbes. To fulfill P nutrient demands, soil microbes could release larger amounts of phosphatase [29]. However, the PO and PEO activities declined following N fertilization. This result could be attributed to the fact that N enrichment usually enhances aboveground litter production by approximately 20% [58]; thus, high litter additions to soil could be one possible mechanism that results in reduced oxidase activity. Another possibility is that these enzymes have a close relationship with fungal diversity and abundance [56], and we also found slightly reduced fungal abundance following N fertilization (Table 4). Additionally, a significant correlation was found between the N, C, and P enzyme activities (Fig 4), implying that the enzyme activities are closely linked (Dai et al., 2019). Notably, the activities of C-cycling enzymes had a direct positive relationship with the activities of N-cycling enzymes (Fig 5), and soil C availability increases N-cycling enzymes [59]. Taken together, our results suggest that soil pH and SOC together determine C-cycling enzyme activities.

### Soil bacteria are more active than fungi in response to soil pH and SOC

Numerous studies have demonstrated that soil pH is the major factor shaping microbial communities [18, 21]. The PLS-PM results of our study revealed that soil pH was the key determining factor affecting soil bacteria, not fungi (Fig 5B). The response of microbial groups under N fertilization varies considerably. It is generally believed that bacteria are more sensitive to pH than fungi [60]. Additionally, Ai et al. (2018) also demonstrated that soil bacteria rather than soil fungi were significantly influenced by the soil pH. Nitrogen accumulation induced by high N inputs can decrease the soil pH [4, 7], resulting in the leaching of calcium and magnesium and aluminum mobilization [49]. Under these conditions, microbial communities face calcium or magnesium shortages or are enriched with aluminum [8], thus influencing soil ecosystem functions. However, other environmental variables can also govern microbial community shifts under N addition. For example, Wang et al. (2018b) demonstrated that shifts in SOC following N addition significantly affected soil bacteria.

N enrichment alters microbial biomass and community structure [6]. In our study, N addition increased the total PLFAs and bacterial abundance and altered the microbial community structure (Table 4; S2 Fig). The bacterial abundance increased and then decreased in response to N addition, i.e., the low N addition increased the bacterial abundance, while the high N addition decreased the bacterial abundance. The enhanced abundance of bacterial communities under the low N fertilization treatment in our study can be ascribed to the following explanations. (i) N addition enhanced the soil TN levels, which then enhanced the SOC contents, and soil bacteria have copiotrophic characteristics [61]. (ii) N addition resulted in a lower substrate C:N ratio that could promote more bacterial growth than fungal growth, as the mean C:N ratio was approximately 4 in bacteria and approximately 10 in fungi [62]. Moreover, we found that the abundance of AMF decreased as a result of N fertilization. The decrease in AMF abundance under N addition in our study is supported by earlier studies showing that N enrichment decreased AMF abundance [63]. Experimental evidences indicated that enhanced N availability as a result of N addition possibly inhibited the allocation of plant C to fine roots, which led to the reduced fungal colonization of roots and population of fungi [64]. In addition, other work demonstrated that the influence of inorganic fertilization on AMF was site specific and linked to the initial soil nutrient conditions [65]. Furthermore, we noticed moderate changes in the relative abundances of microbial groups between the wheat and maize seasons. This seasonal change in the microbial responses under N fertilization can be attributed to different crop types since plant type can substantially impact the adjoining soil and its microbiota [22]. Other studies have indicated that microbial biomass accumulation can change due to root exudate inputs from different vegetation types and plant species that vary in the quality and quantity of the carbon resources they produce [66]. Notably, there were few differences in the bacteria, fungi, and actinomycete abundances between the low N and high N treatments in both seasons. The difference in the N fertilizer rate between these treatments was not large, i.e., the high N treatment received only 23% more N fertilizer than the low N treatment, which may have been the reason for this result. Therefore, a 23% increase in the N rate may not have induced large changes in the soil microbial populations. However, we found that the high N addition slightly decreased the bacterial and actinomycete abundance in our study. A study demonstrated that microbial abundance is a key indicator of soil health and quality and that any reduction in the abundance of microbial populations can hamper soil ecosystem functions [67].

Interestingly, in this study, the MBC was slightly affected; in contrast, the total PLFAs were greatly affected as a result of N addition, particularly in the maize season (Fig 1B; Table 4). These results may be because these two estimations used different components of microbial biomass, while the PLFA assay mainly focused on the active components of biomass [68, 69]. The MBN and MBC contents were measured by flushing the N and C that were liberated from cells as a result of chloroform fumigation, respectively, and the PLFA assay determined the extent to which phospholipids were present in the cell membranes. Consequently, a close link between the microbial biomass determined by the chloroform fumigation method and the microbial biomass determined by the PLFA method cannot always be expected [70]. Additionally, PLFA contents may change even within a particular species based on the quality of the carbon source available [71]. Therefore, distinct results from PLFA analysis and MBC from fumigated soil do not indicate that one of these measurements is ineffective.

## Conclusions

In summary, our results showed that a long-term N addition changed the soil nutrient status. N enrichment also decreased the soil pH; however, the reduction in soil pH was more pronounced in the high N addition treatment. The enzyme activities mainly involved in the cycling of C, N, and P were enhanced as a result of N addition, indicating that under high N conditions, soil microbes tend to produce more enzymes. Additionally, the responses of enzyme activities were consistent in both the wheat and maize crops under N addition; however, the response of microbial biomass varied in the two crop types under N addition. Environmental variables, such as a low soil pH and elevated SOC level, primarily affected the C-cycling enzymes and soil bacteria but had no effect on the soil N-cycling enzymes and fungi. Our study further elucidated the mechanisms behind soil biological changes in response to N addition under wheat-maize rotation in calcareous upland soils. Furthermore, growers in China need to switch from adding large amounts of fertilizer to using low or optimum levels of fertilization. This practice will not only allow growers to achieve high crop productivity but also prevent the adverse effects of agriculture, particularly over fertilization, in the environment.

## Supporting information

S1 Table Effect of crop season, N fertilization and their interaction on extracellular enzyme activities as determined by PERMANOVA analysis. (DOCX)

S2 Table Effect of crop season, N fertilization and their interaction on microbial community composition as determined by PERMANOVA analysis. (DOCX)

S3 Table The direct and indirect relationships between variables. The path coefficients are calculated by PLS-PM after 1000 bootstraps.
(DOCX)

S1 Fig Principal coordinate analysis (PCoA) of enzyme activities of different N treatments for both winter wheat and summer maize seasons. Control = (no N added); low-N = (182 kg ha^-1^ of N); high-N = (225 kg ha^-1^ of N).
(JPG)

S2 Fig Principal coordinate analysis (PCoA) of microbial community composition as determined by phospholipid fatty acids (PLFAs) of different N treatments for both winter wheat and summer maize seasons. Control = (no N added); low-N = (182 kg ha^-1^ of N); high-N = (225 kg ha^-1^ of N).
(JPG)

S1 Dataset The raw data of experiment. (XLSX)
