# Supplementary material for: The responses of extracellular enzyme activities and microbial community composition under nitrogen addition in an upland soil
Source: PLoS One. 2019 Sep 30;14(9):e0223026. doi: 10.1371/journal.pone.0223026 (PMC6768454; doi:10.1371/journal.pone.0223026)
Supplement: S1 Table — (DOCX) [file pone.0223026.s001.docx]

PERMANOVA table of results

| Source | df | SS | MS | Pseudo-F | P(perm) | perms |
| --- | --- | --- | --- | --- | --- | --- |
| Crop season | 1 | 117.68 | 117.68 | 18.29 | 0.001 | 999 |
| N Fertilization | 2 | 114.12 | 57.06 | 8.8681 | 0.001 | 998 |
| Cr × N | 2 | 24.464 | 12.232 | 1.9011 | 0.146 | 998 |
| Res | 18 | 115.82 | 6.4343 |  |  |  |
| Total | 23 | 372.09 |  |  |  |  |

Details of the expected mean squares (EMS) for the model

| Source | EMS |
| --- | --- |
| Crop season | 1*V(Res) + 12*S (Cr) |
| N Fertilization | 1*V(Res) + 8*S (N) |
| Cr × N | 1*V(Res) + 4*S (Cr × N) |
| Res | 1*V(Res) |

Construction of Pseudo-F ratio(s) from mean squares

| Source | Numerator | Denominator | Num.df | Den.df |
| --- | --- | --- | --- | --- |
| Crop season | 1*Cr | 1*Res | 1 | 18 |
| N Fertilization | 1*N | 1*Res | 2 | 18 |
| Cr × N | 1*Cr × N | 1*Res | 2 | 18 |

Estimates of component of variation

| Source | Estimate | Sq.root |
| --- | --- | --- |
| S(N) | 9.2708 | 3.0448 |
| S(Crop) | 6.3282 | 2.5156 |
| S(N × Crop) | 1.4495 | 1.2039 |
| V(Res) | 6.4343 | 2.5366 |
| Total | 23.48 | 9.30 |
