# Supplementary material for: The responses of extracellular enzyme activities and microbial community composition under nitrogen addition in an upland soil
Source: PLoS One. 2019 Sep 30;14(9):e0223026. doi: 10.1371/journal.pone.0223026 (PMC6768454; doi:10.1371/journal.pone.0223026)
Supplement: S2 Table — (DOCX) [file pone.0223026.s002.docx]

PERMANOVA table of results

| Source | df | SS | MS | Pseudo-F | P(perm) | perms |
| --- | --- | --- | --- | --- | --- | --- |
| Crop season | 1 | 18.449 | 18.449 | 7.3129 | 0.001 | 998 |
| N Fertilization | 2 | 23.918 | 11.959 | 4.7404 | 0.001 | 999 |
| Cr × N | 2 | 40.491 | 20.245 | 8.0252 | 0.001 | 998 |
| Res | 18 | 45.409 | 2.5227 |  |  |  |
| Total | 23 | 128.27 |  |  |  |  |

Details of the expected mean squares (EMS) for the model

| Source | EMS |
| --- | --- |
| Crop season | 1*V(Res) + 12*S(Cr) |
| N Fertilization | 1*V(Res) + 8*S(Fe) |
| Cr × N | 1*V(Res) + 4*S(CrxFe) |
| Res | 1*V(Res) |

Construction of Pseudo-F ratio(s) from mean squares

| Source | Numerator | Denominator | Num.df | Den.df |
| --- | --- | --- | --- | --- |
| Crop season | 1*Cr | 1*Res | 1 | 18 |
| N Fertilization | 1*N | 1*Res | 2 | 18 |
| Cr × N | 1*Cr × N | 1*Res | 2 | 18 |

Estimates of components of variation

| Source | Estimate | Sq.root |
| --- | --- | --- |
| S(N) | 1.3272 | 1.152 |
| S(Crop) | 1.1795 | 1.0861 |
| S(N × Crop) | 4.4307 | 2.1049 |
| V(Res) | 2.5227 | 1.5883 |
| Total | 9.46 | 5.93 |
