# Supplementary material for: The responses of extracellular enzyme activities and microbial community composition under nitrogen addition in an upland soil
Source: PLoS One. 2019 Sep 30;14(9):e0223026. doi: 10.1371/journal.pone.0223026 (PMC6768454; doi:10.1371/journal.pone.0223026)
Supplement: S3 Table — The path coefficients are calculated by PLS-PM after 1000 bootstraps. (DOCX) [file pone.0223026.s003.docx]

The direct and indirect relationships between variables. The path coefficients are calculated by PLS-PM after 1000 bootstraps.

| Wheat |  |  |  |  | Maize |  | |  | |  | |  |
| --- | --- | --- | --- | --- | --- | --- | --- | --- | --- | --- | --- | --- |
| Relationships | Direct | Indirect | Total |  | Relationships | Direct | | Indirect | | Total | |  |
| N addition -> pH | -0.67 | 0 | -0.67 |  | N addition -> pH | | -0.8 | | 0 | | -0.8 | |
| N addition -> N | 0.72 | 0.13 | 0.86 |  | N addition -> N | | 0.69 | | 0.24 | | 0.93 | |
| N addition -> SOC | 0.79 | -0.05 | 0.73 |  | N addition -> SOC | | 0.56 | | 0.19 | | 0.76 | |
| N addition -> Bacteria | 0 | 0.47 | 0.47 |  | N addition -> Bacteria | | 0 | | 0.58 | | 0.58 | |
| N addition -> Fungi | 0 | -0.02 | -0.02 |  | N addition -> Fungi | | 0 | | -0.10 | | -0.10 | |
| N addition -> C enzymes | 0 | 0.70 | 0.70 |  | N addition -> C enzymes | | 0 | | 0.60 | | 0.60 | |
| N addition -> N enzymes | 0 | 0.64 | 0.64 |  | N addition -> N enzymes | | 0 | | 0.76 | | 0.76 | |
| pH -> N | -0.20 | 0 | -0.20 |  | pH -> N | | -0.30 | | 0 | | -0.30 | |
| pH -> SOC | 0.08 | 0 | 0.08 |  | pH -> SOC | | -0.24 | | 0 | | -0.244 | |
| pH -> Bacteria | -0.33 | 0.15 | -0.18 |  | pH -> Bacteria | | -0.92 | | 0.07 | | -0.88 | |
| pH -> Fungi | -0.43 | 0.07 | -0.35 |  | pH -> Fungi | | -0.81 | | 0.24 | | -0.56 | |
| pH -> C enzymes | -0.52 | 0.28 | -0.24 |  | pH -> C enzymes | | 0.06 | | -0.44 | | -0.38 | |
| pH -> N enzymes | -0.01 | -0.26 | -0.27 |  | pH -> N enzymes | | -0.48 | | -0.03 | | -0.51 | |
| N -> SOC | 0 | 0 | 0 |  | N -> SOC | | 0 | | 0 | | 0 | |
| N -> Bacteria | -0.41 | 0 | -0.41 |  | N -> Bacteria | | -0.34 | | 0 | | -0.34 | |
| N -> Fungi | -0.37 | 0 | -0.37 |  | N -> Fungi | | -0.72 | | 0 | | -0.72 | |
| N -> C enzymes | 0 | 0.33 | 0.33 |  | N -> C enzymes | | 0 | | -0.16 | | -0.16 | |
| N -> N enzymes | -0.19 | 0.25 | 0.06 |  | N -> N enzymes | | 0.17 | | 0.06 | | 0.23 | |
| SOC -> Bacteria | 0.82 | 0 | 0.82 |  | SOC -> Bacteria | | 0.12 | | 0 | | 0.12 | |
| SOC -> Fungi | 0.01 | 0 | 0.01 |  | SOC -> Fungi | | -0.10 | | 0 | | -0.10 | |
| SOC -> C enzymes | 0.75 | -0.37 | 0.38 |  | SOC -> C enzymes | | 0.71 | | 0.01 | | 0.72 | |
| SOC -> N enzymes | 0 | 0.52 | 0.52 |  | SOC -> N enzymes | | 0 | | 0.32 | | 0.32 | |
| Bacteria -> C enzymes | -0.44 | 0 | -0.44 |  | Bacteria -> C enzymes | | 0.21 | | 0 | | 0.21 | |
| Bacteria-> N enzymes | 0.15 | -0.46 | -0.30 |  | Bacteria-> N enzymes | | -0.12 | | 0.09 | | -0.03 | |
| Fungi -> C enzymes | -0.39 | 0 | -0.39 |  | Fungi -> C enzymes | | 0.12 | | 0 | | 0.12 | |
| Fungi -> N enzymes | 0.07 | -0.40 | -0.33 |  | Fungi -> N enzymes | | -0.12 | | 0.05 | | -0.06 | |
| CE -> N enzymes | 0.94 | 0 | 0.94 |  | CE -> N enzymes | | 0.44 | | 0 | | 0.44 | |
