# Supplementary figures and images for: The responses of extracellular enzyme activities and microbial community composition under nitrogen addition in an upland soil
Source: PLoS One. 2019 Sep 30;14(9):e0223026. doi: 10.1371/journal.pone.0223026 (PMC6768454; doi:10.1371/journal.pone.0223026)

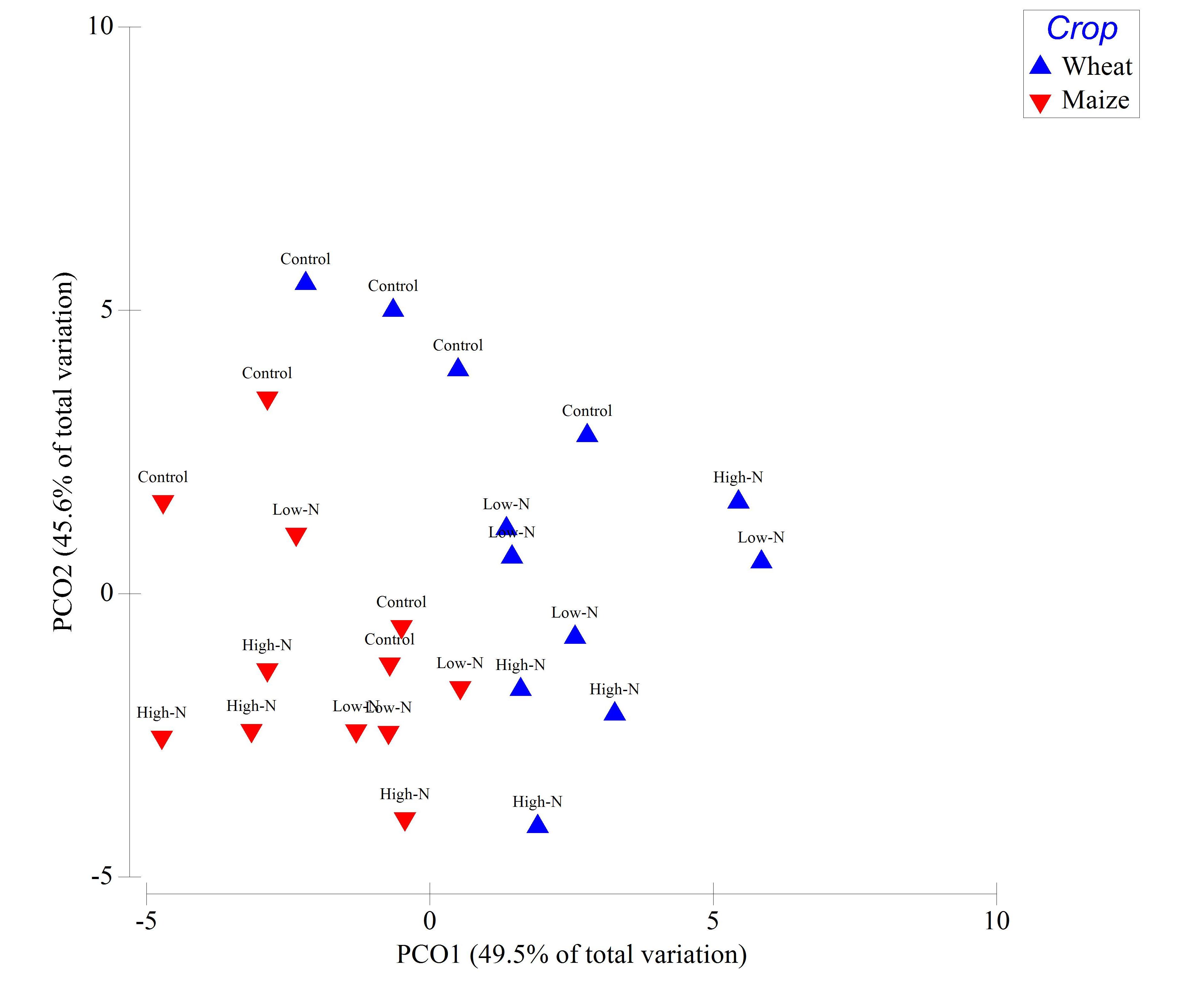

Supplement: S1 Fig — Control = (no N added); low-N = (182 kg ha-1 of N); high-N = (225 kg ha-1 of N). (JPG) [file pone.0223026.s004.jpg]

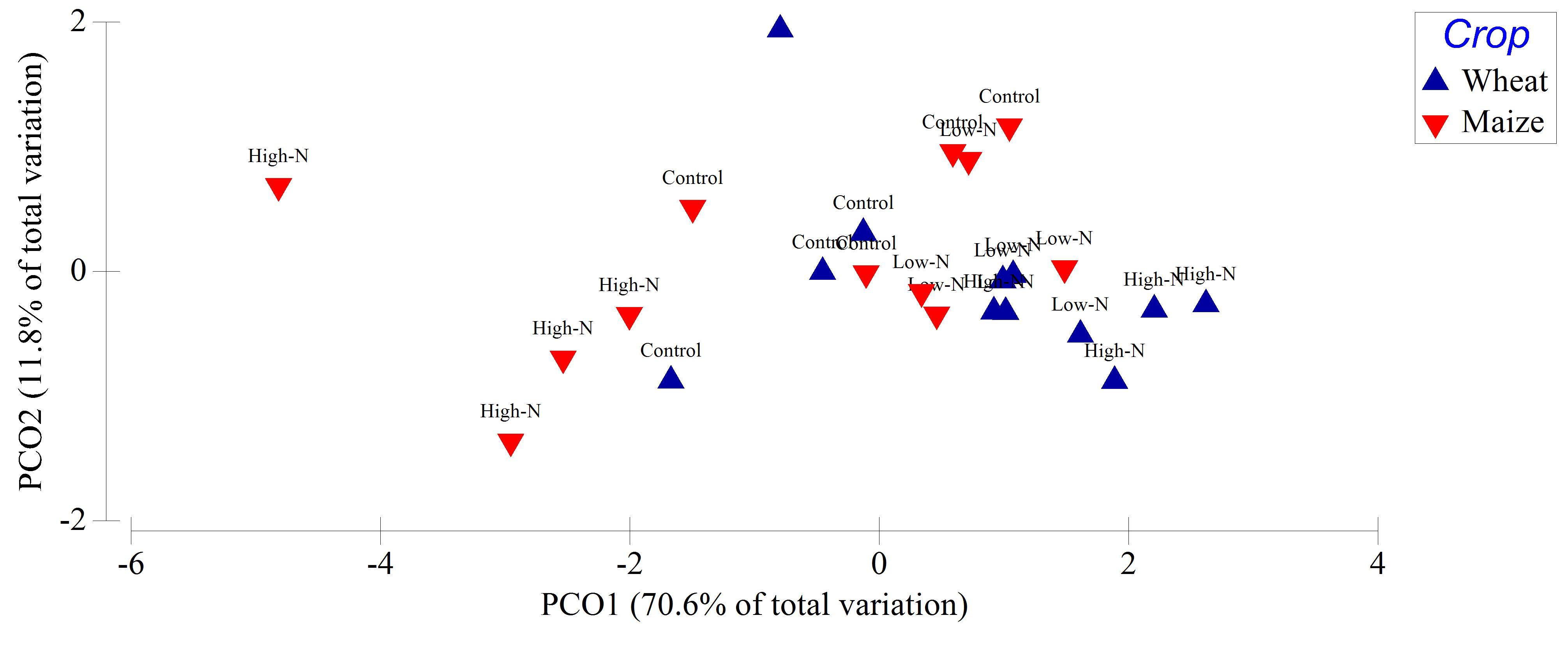

Supplement: S2 Fig — Control = (no N added); low-N = (182 kg ha-1 of N); high-N = (225 kg ha-1 of N). (JPG) [file pone.0223026.s005.jpg]
